# ASO Author Reflections: The Peritoneum is an Active Immunological Compartment

**DOI:** 10.1245/s10434-021-10036-8

**Published:** 2021-05-03

**Authors:** Ebbe Billmann Thorgersen, Kjersti Flatmark

**Affiliations:** 1 Department of Gastroenterological Surgery, Oslo University Hospital, The Radium Hospital, Oslo, Norway; 2 Department of Immunology, Oslo University Hospital Rikshospitalet, Oslo, Norway; 3 Faculty of Medicine, University of Oslo, Oslo, Norway; 4 Department of Tumor Biology, Oslo University Hospital, The Radium Hospital, Oslo, Norway

## Past

Cytoreductive surgery (CRS) and hyperthermic intraperitoneal chemotherapy (HIPEC) is established as standard-of-care treatment for selected patients with resectable peritoneal surface malignancies, such as peritoneal metastasis from colorectal cancer (PM-CRC).^1^ Although CRS-HIPEC may improve overall survival, the majority of patients experience disease relapse and new treatment options are needed.^2^ Anticancer therapy exploiting immune mechanisms, including inflammation, could potentially be an interesting strategy in compartmentalized treatment in the peritoneal cavity;^3^ however, little is known about local and systemic immune response after major abdominal surgery.

## Present

In the recent work ‘Increased Local Inflammatory Response to MOC31PE Immunotoxin After Cytoreductive Surgery and Hyperthermic Intraperitoneal Chemotherapy’, a highly compartmentalized, broad, and substantial intraperitoneal inflammatory cytokine response was identified after CRS-HIPEC compared with a modest systemic inflammatory response.^4^ Several cytokines exhibited distinct time-dependent release patterns, some with very high intraperitoneal peak levels. The response was further enhanced in patients who received treatment with MOC31PE immunotoxin targeting epithelial cell adhesion molecule. Of particular interest, several of these cytokines, including the classical proinflammatory cytokine interleukin-6, and the T-cell stimulator interferon (IFN)-γ and its companion IFN-induced protein-10, are involved in processes related to immune activation with a potential anticancer effect, such as immunogenic cell death.

## Future

Interpretation of these findings is challenging because of the dualistic nature of inflammation as both a promoter and inhibitor of cancer initiation and progression.^5^,^6^ It is further complicated by temporal issues, where the chronic low-grade inflammation in a tumor microenvironment will differ from acute inflammation caused by surgical intervention. The peritoneum seems to be a very active immunological ‘organ’, and improved knowledge of peritoneal biology, in particular of humoral and cellular immune mechanisms, would likely improve our understanding of peritoneal cancer dissemination and its treatment. Analysis of immune cells in tissues and peritoneal fluid before and after CRS-HIPEC is a logical next experimental step. The knowledge could be essential to precisely tailor-make new immunomodulating treatment regimens as a supplement to CRS-HIPEC for PM-CRC to improve long-term oncological outcomes.
